# Omsk Hemorrhagic Fever Virus and Powassan Virus Infections at Different Times After Immunization with the TBE Vaccine in In Vivo Experiments

**DOI:** 10.3390/ijms27125435

**Published:** 2026-06-16

**Authors:** Viktoria Kuchina, Ksenia Tuchynskaya, Anastasia Rogova, Galina Karganova

**Affiliations:** 1 Laboratory of Biology of Arboviruses, Chumakov Federal Scientific Center for Research and Development of Immune-and-Biological Products of Russian Academy of Sciences (Institute of Poliomyelitis), Moscow 108819, Russia; villarionova1@gmail.com (V.K.); kseniya-tuchka@mail.ru (K.T.); mfz106@yandex.ru (A.R.); 2 Faculty of Biology, Lomonosov Moscow State University, Moscow 119234, Russia; 3 Institute for Translational Medicine and Biotechnology, Sechenov First Moscow State Medical University, Moscow 119991, Russia

**Keywords:** orthoflavivirus, TBEV, OHFV, POWV, TBE vaccine, cross-reactive antibodies

## Abstract

Ranges of distribution of some orthoflaviviruses, including the Omsk hemorrhagic fever virus (OHFV) and the Powassan virus (POWV), have been identified in the range endemic for the tick-borne encephalitis virus (TBEV). In contrast to TBEV, no registered vaccine against OHFV and POWV is currently available. Nevertheless, recent studies have indicated that the anti-TBEV vaccine may offer partial protection against other orthoflaviviruses. The present study assesses OHFV and POWV infection in an experimental model on BALB/c mice one, three- and twelve-months post-immunization with inactivated TBE vaccine. The TBE vaccine was shown to provide long-term protection against OHFV and partial protection against POWV, even in the absence of specific antibodies, and without indications of antibody-dependent enhancement of infection. The neutralizing antibody titer for OHFV in vaccinated animals before and after the challenge with OHFV was similar to the titer of antibodies against the TBEV vaccine strain. Following immunization with anti-TBEV vaccine and subsequent inoculation of POWV, the levels of neutralizing antibodies against the TBEV vaccine strain was observed to be higher compared to those targeting POWV, especially during the initial phase of infection. Such cross-reactive antibodies have potential to pose a significant diagnostic challenge in cases where an infection is occurring simultaneously with an immune response to another virus.

## 1. Introduction

Orthoflaviviruses are enveloped viruses belonging to the family *Flaviviridae*. The genome of orthoflaviviruses is represented by a positive single-stranded RNA with one open reading frame that encodes three structural proteins (C, capsid; prM, pre-membrane; E, envelope) and seven non-structural proteins (NS1, NS2A, NS2B, NS3, NS4A, NS4B, and NS5). Most orthoflaviviruses are arboviruses, encompassing a range of viruses that pose a significant risk to human health. These include the *Orthoflavivirus denguei* (dengue virus—DENV), *Orthoflavivirus flavi* (yellow fever virus—YFV), *Orthoflavivirus zikaense* (Zika virus—ZIKV), *Orthoflavivirus japonicum* (Japanese encephalitis virus—JEV), *Orthoflavivirus nilense* (West Nile virus—WNV), etc., transmitted by mosquitoes, and *Orthoflavivirus encephalitidis* (tick-borne encephalitis virus—TBEV), *Orthoflavivirus omskense* (Omsk haemorrhagic fever virus—OHFV), *Orthoflavivirus powassanense* (Powassan virus—POWV), *Orthoflavivirus kyasanurense* (Kyasanur Forest disease virus—KFDV), etc., transmitted by ticks [1].

TBEV is the most common tick-borne orthoflavivirus. Approximately 10,000–15,000 cases of tick-borne encephalitis (TBE) are documented annually [2]. Serological prevalence studies demonstrate that inapparent infections account for 70% to 95% of all TBEV infections, depending on the region [3,4]. The incubation period of TBE is estimated to range from 7 to 21 days. The manifestations of the first phase of the disease include elevated body temperature, accompanied by headache, fatigue, nausea and vomiting. In most cases, complete recovery is achieved following the first phase. In approximately one-third of patients, the first phase is followed by the second phase. It is accompanied by fever and neurological symptoms as the virus affects the central nervous system (CNS), which can cause meningitis, meningoencephalitis, meningoencephalomyelitis, or meningoencephaloradiculitis [5,6]. Protracted convalescence is frequently observed for severe illness; in some instances the infection becomes chronic [7,8]. There is significant variation in the case fatality rate of TBE: it ranges from 2% to 20%, contingent on a multitude of factors associated with both the properties of the virus and the patient’s characteristics [9,10,11]. Five inactivated whole-virion vaccines for TBE prevention have been approved for use: two European, two Russian, and one Chinese vaccine [12,13,14]. All TBE vaccines are available in adult and pediatric versions. Approximately 3 million individuals in Russia receive vaccinations or revaccinations on an annual basis. This fact is indicative of the fairly high level of TBEV seropositivity among the population in Russia [15,16].

TBE infection in humans may confer lifelong immunity due to a high T- and B-cell response [10,17]. Immunization with an inactivated whole-virion vaccine against TBEV was also shown to activate both CD4+ and CD8+ T-cells. However, the profile of these cells and their activation degree vary compared to those observed for natural infection [17,18,19]. It is imperative to adhere to a regular revaccination schedule to ensure the maintenance of protective antibody (Ab) titers and the subsequent formation of long-lived B- and CD4+ memory T-cells [20].

Other orthoflaviviruses were found in regions endemic for TBE. They include OHFV in Western Siberia, POWV in the Russian Far East, JEV in the Russian Far East and China, and WNV in Europe and the European part of Russia [10,21,22,23]. There is therefore geographic overlap in the range of these different orthoflaviviruses. Furthermore, individuals vaccinated against TBEV or exposed to it may travel to regions endemic for other orthoflaviviruses. It means that simultaneous or sequential infection with several viruses, or infection with another orthoflavivirus while immunized with the TBE vaccine, may occur [10,24].

In addition to the Western Siberian regions of the Russian Federation, OHFV has been detected in Kazakhstan, where TBE cases have also been reported [25,26]. In 1988–1997, 165 cases of Omsk hemorrhagic fever (OHF) infection were reported in Russia, but this incidence might be incomplete, since asymptomatic cases or cases with a mild course have probably been unreported. Strength of the immunity to OHFV in endemic areas is also unknown because of the lack of appropriate test systems [27]. Hence, the current statistics on the incidence of OHF are unclear. The infection is known to be seasonal; along with ticks, the sources of infection include muskrats (people get infected when cutting their carcasses) [27].

OHF infection has a shorter incubation period (3–7 days) compared to TBE and is usually associated with internal organ and/or skin hemorrhages. Within one or two weeks, 50–70% of patients recover, while others enter the second phase of the disease. This phase also lasts one or two weeks and is characterized by prolonged fever, hemorrhages, and the emergence of encephalitic symptoms [27]. As a rule, patients usually recover completely, but there are cases of persistent long-term complications. The mortality of OHF was shown to vary between 0.4% and 2.5% [27].

POWV is distributed in Canada, the United States, and the Russian Far East [28]. POWV is a neurotropic virus, and the clinical picture of infection is very similar to that of TBE infection. The mortality rate of severe POWV infection is up to 10%, but these statistics are very approximate because of the lack of specific test systems to detect anti-POWV antibodies [29]. No vaccine against POWV is currently registered, but its development is underway [30,31].

TBEV has several subtypes. The most common ones are Far Eastern, Siberian, and European subtypes, which are named after the respective regions where they are distributed [10]. OHFV has three subtypes (1, 2, and 3) [32], and POWV has two lineages: lineage 1—POWV in Russia and North America; lineage 2—the deer tick virus circulating in Canada and the United States [28,33]. OHFV and POWV are members of the TBEV serocomplex [34,35]. Phylogenetic analysis indicates that, with allowance for the evolutionary characteristics of OHFV, it is closely related to TBEV. Therefore, it has been proposed that TBEV and OHFV should be classified as a single species [36]. Among them, POWV is most distinct antigenically and phylogenetically [37]. The similarity of the surface proteins of viruses belonging to the same serocomplex leads to cross-reactive Ab response. In experimental subcutaneous infection of non-human primates, TBE vaccination protected the animals against hemolytic syndrome during OHFV infection. However, it ensured no protection against viral entry into the brain or visceral organs [38]. Moreover, TBE vaccination protected the primates against viremia and the presence of viral RNA in organs during POWV infection [39]. In experiments on mice conducted two weeks after double-dose immunization with an inactivated lyophilized TBE vaccine, protection against mortality was demonstrated for OHFV infection, while no clear protection was observed in the cases of POWV infection [40,41]. A comparable outcome was attained in an in vitro experiment with sera from vaccinated mice. These sera exhibited elevated protective titers in the neutralization test against OHFV [40,41,42], but not POWV [40]. When studying the serum samples from people vaccinated with the European vaccine against TBE, as well as people after TBE infection, it was shown that the titers of neutralizing antibodies (NAbs) against TBEV and OHFV were approximately at the same level. However, only a subset of sera exhibited neutralizing activity against POWV, with generally low titers, particularly in samples obtained from vaccinated individuals [43].

For mosquito-borne orthoflaviviruses, both within the same serocomplex (DENV types 1, 2, 3 and 4) and between different serocomplexes (DENV and ZIKV), cross-reactive Abs may both play a protective role during infection with closely related orthoflaviviruses and cause antibody-dependent enhancement of infection (ADE) [44,45]. Infection is enhanced by secondary infection with DENV of a different subtype. Several mechanisms of ADE have been described in the literature [46,47,48], but the most widely studied one is the viral entry into the cell through the interaction between the Fc part of Ab and the Fcγ receptor on the cell surface [49]. This pathway of viral entry into the cell occurs in the presence of non-neutralizing Abs or Abs at sub-neutralizing concentrations. Therefore, the course of infection must be studied in the context of pre-existing antibodies specific to various orthoflaviviruses in order to gain a more profound understanding of the nature and mechanisms of ADE. It may also be useful in predicting disease severity.

There is a large body of research on ADE between mosquito orthoflaviviruses of different serocomplexes in vitro and in vivo, including experiments in rhesus macaques [44,50,51,52,53,54,55]. ADE has also been shown to be possible after vaccination with inactivated and recombinant vaccines [56,57]. However, the issue of ADE is poorly understood for tick-borne orthoflaviviruses. The level of post-vaccination anti-TBEV antibodies is known to decline with time after vaccination, and the Ab spectrum may also change [58,59]. It may affect both the effectiveness of protection in the case of infection with another orthoflavivirus and increase the probability of ADE.

The present study aims to provide analysis of the infection caused by OHFV and POWV in BALB/c mice that are both vaccinated and unvaccinated against TBEV, with a particular focus placed on the long-term post-vaccination immune response induced by the TBE vaccine.

## 2. Results

### 2.1. Characteristics of the Orthoflaviviruses Studied

Even virus strains belonging to the same subtype can vary significantly in pathogenicity, particularly in relation to their neurovirulence and neuroinvasiveness in vivo [14,60,61]. Some orthoflavivirus strains are characterized by nonlinear mortality during virus titration in mice [62,63,64], which introduces some complications in the use of Reed–Muench and Kärber methods for determining LD_50_. A similar trend was observed in our study for POWV. Even at high doses, it did not cause 100% mortality in BALB/c mice. Nevertheless, for comparative assessment of virus virulence, we calculated the conventional LD_50_ value using the Kärber method.

The neuroinvasiveness index is a parameter assessed in the study denoting the degree of pathogenicity, with a higher index corresponding to lower pathogenicity. The neuroinvasiveness index was defined as the difference between the logarithms of the viral plaque-formation units (PFUs) in PEK cell culture (log(PFU/mL)) and the virus titer calculated as 50% lethal dose (LD_50_) after subcutaneous infection of BALB/c mice (log(LD_50_/mL)): log(PFU/mL) − log(LD_50_/mL). As demonstrated in Table 1, the viruses used in the study exhibited divergent degrees of neuroinvasiveness. The highest neuroinvasiveness was observed for TBEV (1.0) and OHFV (1.6), while POWV was characterized by the lowest pathogenicity in experimental infection (2.8).

### 2.2. The Effect of the Age of BALB/c Mice on Infection Caused by TBEV, OHFV, and POWV

Patient age was found to be a contributing factor to the severity of diseases caused by orthoflaviviruses [65,66,67]. Our study examined the effect of age on the course of infection in an experimental mouse model. The study included three age groups of mice: young (14-week-old), mature (24-week-old), and old (64-week-old) [68]. The mice were subcutaneously infected with viruses at a dose of 100LD_50_. The assessment of mortality and morbidity was based on weight loss and clinical manifestations of the disease. The brains of surviving animals were examined by qRT-PCR to detect viral RNA.

**Figure 1:**
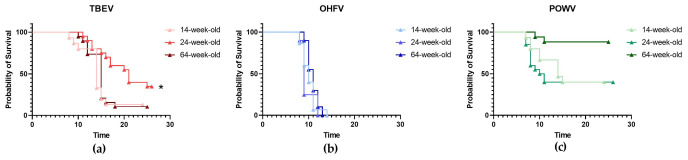
The survival rate of mice of different ages following subcutaneous infection with TBEV (**a**), OHFV (**b**), and POWV (**c**) at doses of 100LD_50_, *n* = 15–20. Statistical differences were determined using the log-rank test (* *p* < 0.05).

The survival rate and the median lifespan of mature 24-week-old mice infected with TBEV (EK-328) were statistically higher than in young and old (14- and 64-week-old) mice (Figure 1, Table 2). As illustrated in Figure 1b, the infection of mice with OHFV resulted in 100% mortality within 13 days of infection, with no discernible impact on mortality based on age. Mortality and morbidity following POWV infection showed the greatest differences depending on the age of the mice compared to TBEV and OHFV. For young 14-week-old and mature 24-week-old mice infected with POWV, the mortality rate and the percentage of mice in whose CNS the virus had been detected in the late stages of infection (24–28 days post-infection) were approximately the same (60–70%). Meanwhile, the morbidity of young mice was 60%, while it rose to 90% for mature mice. In older mice, the mortality after POWV infection with the same virus dose fell to 12%, but the morbidity and presence of the virus in the CNS 24–28 days post-infection were observed in 94% and 82% of mice, respectively (Figure 1c, Table 2). The morbidity was found to be significantly higher in old mice compared to young mice. Hence, older mice died significantly less often when infected with POWV; despite this, the ability of the virus to penetrate the CNS and cause disease without fatal consequences increased, which may indicate that the infection became chronic.

The dynamics and spectrum of induced antibodies are important characteristics of viral infection. We found that the titers of NAbs against the viruses under study changed with the age of the surviving mice by day 28 post-infection with TBEV (EK-328) and POWV, when the humoral response had already been formed. It was impossible to compile such statistics for mice infected with OHFV, since all the animals died by day 13 post-infection.

**Figure 2:**
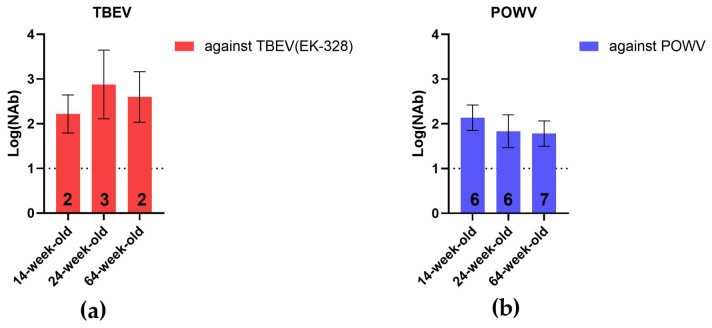
Mean geometric NAbs titers on day 28 post-infection with TBEV (EK-328) (**a**) or POWV (**b**) against TBEV (EK-328) and POWV, respectively, of 14-, 24-, and 60-week-old mice. The number of samples is indicated at the bottom of each column. The dotted line indicates the estimated minimum protective titer of NAbs and the lower limit of NAb detection in PRNT_50_. The spread is represented by the mean value ± SD.

The presence of NAbs against the virus used for the experiment was detected at 28 days post-infection in all the mice infected with TBEV (EK-328) and POWV. Statistically significant differences in NAb titers were observed in neither young, adult, nor old mice (Figure 2a).

The results of the experiment suggest that the age of surviving BALB/c mice does not affect the NAb titers at 28 days post-infection with POWV or TBEV.

### 2.3. Post-Vaccination Immune Response Against TBEV, OHFV, and POWV at Different Time Points After Immunization with the TBE Vaccine

The NAb titers and seroconversion in the mouse sera were analyzed against TBEV vaccine strain Sofjin and the challenge viruses. The analyses were performed one month after a single vaccine dose, and one month, three months, and one year after the final dose in double-dose vaccination against TBEV (Figure 2).

As one might expect, the number of seropositive sera was found to be low (20–40%) one month after a single immunization, both against the TBEV vaccine strain and OHFV. This finding is consistent with the results of a previous study [58]. Among seropositive animals, NAb titers against OHFV were approximately at the same level as for the TBEV vaccine strain.

**Figure 3:**
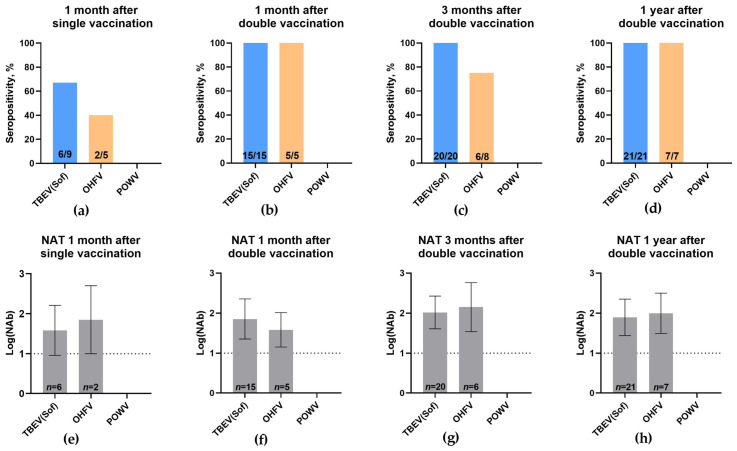
The seropositivity rate and the geometric mean NAb titers in the sera of vaccinated against TBEV mice of different ages. The upper row of graphs (**a**–**d**) shows the number of seropositive sera (*n* = 15–21 sera for determining the NAb titer to TBEV (Sofjin) and *n* = 5–8 for determining the NAb titers to OHFV, POWV). The bottom row of graphs (**e**–**h**) shows the geometric mean NAb titers. The dotted line indicates the estimated minimum protective titer NAbs and the lower limit of NAb detection in PRNT_50_. The data are presented as mean ± SD.

Following the double-dose vaccination of animals with a 14-day interval, a high number of seropositive sera was observed for both TBEV strain Sofjin (100%) and OHFV (100%) one month post-vaccination. The titers of cross-reactive NAbs against OHFV were found to be equivalent to those of the TBEV vaccine strain. The titers of cross-reactive NAbs against POWV were not detected one month after single or double vaccination (Figure 3a,b,e,f).

Three months and one year after receiving two doses of the vaccine, the number of seropositive sera remained high against both TBEV and OHFV. Meanwhile, the NAb titers against TBEV (strain Sofjin) and OHFV remained higher than the putative protective titer of 1 log even one year after double-dose vaccination. The NAb titers against OHFV, as well as one month after double-dose immunization with the TBE vaccine, was equal to the NAb titer against the TBEV strain Sofjin. Three months and one year after receiving two doses of the TBE vaccine, no NAbs against POWV were detected in any of the study groups (Figure 3c,d,g,h).

### 2.4. Protective Efficacy of the TBE Vaccine Against the Orthoflaviviruses Under Study

The protective effect of the TBE vaccine was assessed based on the morbidity and mortality of mice, as well as the presence of the virus in the brains of surviving animals by 24–28 days post-infection of the mice. Subsequently, groups of mice infected with TBEV (EK-328), OHFV, and POWV will be considered based on the time of infection after the last vaccination.

#### 2.4.1. The Survival Rate of Mice Infected with TBEV, OHFV, and POWV One Month After Single- and Double-Dose Immunization with the TBE Vaccine

**Figure 4:**
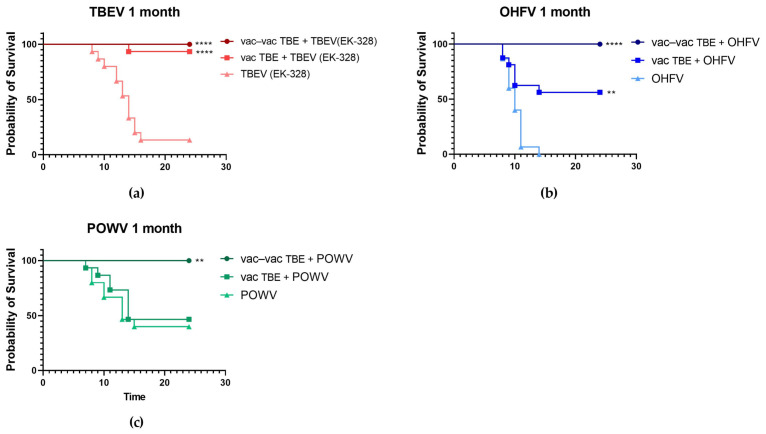
The survival curves of mice infected with TBEV (EK-328) (**a**), OHFV (**b**), and POWV (**c**) at doses of 100LD_50_ one month after single- or double-dose vaccination, *n* = 15. The statistical significance of the mortality data was determined using the log-rank test (** *p* < 0.01; **** *p* < 0.0001).

The survival, absence of morbidity, and absence of virus in the CNS by 24–28 days post-infection were observed in 93% of animals in the group infected with the EK-328 virus one month following single-dose immunization with the TBE vaccine compared to the unvaccinated control group, where the survival rate was 13% and the percentage of healthy animals was 0 % (Figure 4a, Table 3). These findings are indicative of high vaccine protection even after single-dose immunization, despite the low seropositive rate (Figure 3a). One month after administration of two doses of the TBE vaccine, there was 100% survival, no incidence of the disease, and no virus in the CNS by 24–28 days post-infection.

In OHFV-infected mice, one month after single-dose immunization with the TBE vaccine, the survival rate and the percentage of healthy animals was 53%. In the control group animals, which did not receive the vaccine, the mortality rate was 100%, indicating that the vaccine provides partial protection against OHFV even after a single dose (Figure 4b, Table 3). The administration of two doses of the TBE vaccine resulted in the complete protection of mice from the disease and death when infected with OHFV one month after the last immunization (Figure 4b). Concurrently, OHFV was not detected in the CNS of surviving animals by 24 days post-infection (Table 3).

One month after a single-dose immunization with the TBE vaccine in the group infected with POWV, the survival rate and the percentage of healthy mice were approximately equivalent to those in the control group animals that had not been vaccinated. This finding indicates that the single dose of the TBE vaccine does not ensure protection against POWV. In the event of infection with POWV occurring one month after administration of two doses of the TBE vaccine, 100% survival was observed, with no incidence of the disease and no virus detected in the CNS (Figure 4c, Table 3). This outcome was achieved despite the complete absence of antibodies against POWV after double-dose immunization with the TBE vaccine (Figure 3).

#### 2.4.2. NAb Titers After Infection with TBEV, OHFV, and POWV, One Month After Single- and Double-Dose Vaccination Against TBEV

The booster antibody response, resulting in an increase in the NAb titer post-infection, is a significant characteristic of the immune response. A comparison was made of NAb titers before infection, on the second day, and 24–28 days post-infection against the TBEV vaccine strain (Sofjin) and the challenge viruses. In our experiment, the booster response was observed in none of the study groups.

**Figure 5:**
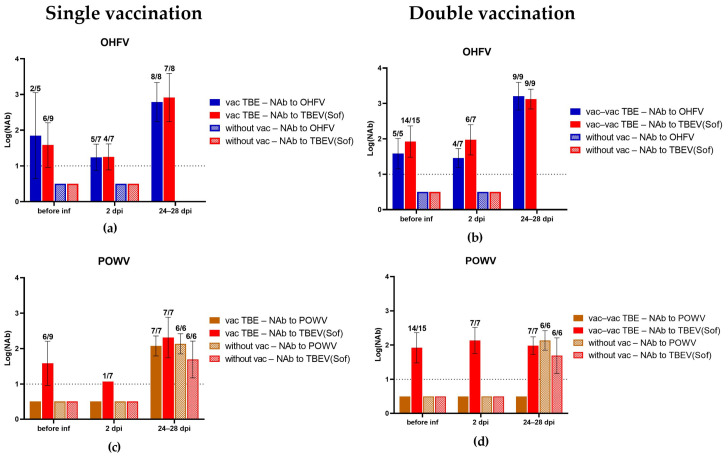
Geometric mean titers of NAbs in sera of seropositive mice before and after infection with OHFV (**a**,**b**) and POWV (**c**,**d**) one month after single-dose (**a**,**c**) and double-dose (**b**,**d**) immunization with the TBE vaccine before and after infection with OHFV and POWV. Solid columns indicate vaccinated groups; shaded columns indicate unvaccinated control groups. Unvaccinated mice infected with OHFV do not have a point at 24–28 days post-infection because of the high mortality before the end of the experiment. The number of subjects in each group is as follows: *n* = 5–7 for vaccinated mice; *n* = 3 for unvaccinated mice before infection and at 2 dpi; *n* = 7 for unvaccinated mice infected with POWV. The number of seropositive sera out of the total number of sera tested is indicated above each column. The dotted line indicates the presumed minimum protective titer of NAbs and the lower limit of NAb detection in PRNT_50_. In sera with NAb titers below 1 log, the conditional titer is taken as 0.5 log. The data are presented as mean ± SD.

The NAb dynamics after a single vaccination and subsequent infection were found to be comparable for all the viruses under study, both against the vaccine strain TBEV and the virus used for the infection (Figure 5a,c).

In the group infected with OHFV after two vaccinations, the titer of NAbs against the TBEV strain Sofjin and OHFV was approximately the same at all time points. A downward trend in NAbs against OHFV was observed on day 2 post-infection (Figure 5b).

In the group infected with POWV after receiving two vaccine doses, NAb titer to the vaccine TBEV strain remained at a high level across all time points. Only this group showed a slight increase in NAbs against the vaccine strain on day 2 post-infection with POWV. NAbs against POWV after two doses of the TBE vaccine were not detected at any time point, including day 28 (Figure 5d).

#### 2.4.3. The Survival Rate of Mice Infected with TBEV, OHFV, and POWV Three Months After Vaccination Against TBE

**Figure 6:**
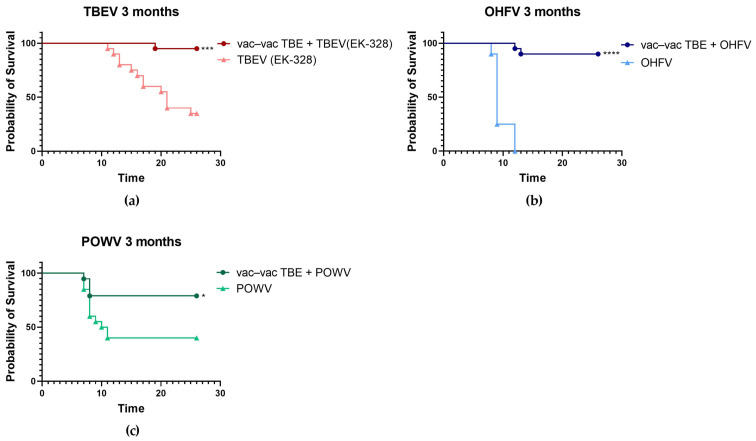
The survival curves of mice infected with TBEV (EK-328) (**a**), OHFV (**b**), and POWV (**c**) at doses of 100LD_50_ three months after double-dose immunization with the TBE vaccine, *n* = 19–20. The statistical significance of the mortality data was determined using the log-rank test (* *p* < 0.05; *** *p* < 0.001; **** *p* < 0.0001).

Three months after receiving two doses of the TBE vaccine, the group infected with TBEV (EK-328) was characterized by significantly higher survival and lower morbidity rates than the unvaccinated control group. Simultaneously, the virus was detected in the CNS at 24–28 days post-infection in a mere 10% of the surviving vaccinated animals, whereas this figure was 100% in the control group (Figure 6a, Table 4).

Three months after two TBE vaccine doses, the survival rate in the group infected with OHFV was 90%, vs. 0% survivors in the unvaccinated control group (Figure 6b, Table 4). Consequently, even three months after the last immunization, the TBE vaccine exhibited a high efficacy against OHFV.

Three months after two doses of the TBE vaccine, the survival and health rates in the group of vaccinated mice infected with POWV were found to be significantly higher than those in the unvaccinated control group (79% vs. 40% and 10%, respectively) (Figure 6c, Table 4). This is indicative of partial protection by the vaccine against POWV, despite the absence of neutralizing antibodies against POWV.

#### 2.4.4. NAb Titers After Infection with TBEV, OHFV, and POWV Three Months After Vaccination Against TBE

Three months after vaccination with two doses of TBE vaccine against infection by studied orthoflaviviruses did not display a booster antibody response, similar to the observations made one month post-vaccination.

**Figure 7:**
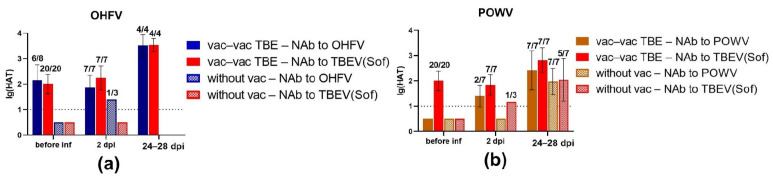
Mean geometric titers of NAbs in sera of seropositive mice against OHFV (**a**) and POWV (**b**) three months after double-dose immunization with the TBE vaccine before and after infection with OHFV and POWV. The vaccinated groups are indicated by solid columns; the unvaccinated control groups by shaded columns. Unvaccinated mice infected with OHFV do not have a point at 24–28 day post-infection because of the high mortality before the end of the experiment. The number of subjects in each group is as follows: *n* = 7 in the vaccinated group; *n* = 3 in the unvaccinated group before infection and at 2 days postinfection; *n* = 7 in the unvaccinated group at 24–28 day post-infection. The number of seropositive sera out of the total number of sera tested is indicated above each column. The dotted line indicates the presumed minimum protective titer of NAbs and the lower limit of NAb detection in PRNT_50_. In sera with NAb titers below 1 log, the conditional titer is taken as 0.5 log. The data are presented as mean ± SD.

In the cohort of mice that had received a double-dose vaccination and were subsequently infected with OHFV, NAb titers against the vaccine TBEV strain and OHFV exhibited comparable dynamics (Figure 7a).

In the group infected with POWV, NAbs were detected in vaccinated mice before infection and on day 2 post–infection only against the TBEV vaccine strain Sofjin. Furthermore, in one serum sample, NAbs against POWV were identified two days post-infection (Figure 7b). In contrast to the mice infected one month after two vaccinations with the TBE vaccine, the NAb titers to POWV in this group had increased by 24–28 days post–infection.

#### 2.4.5. The Survival Rate of Mice Infected One Year After Vaccination Against TBE

**Figure 8:**
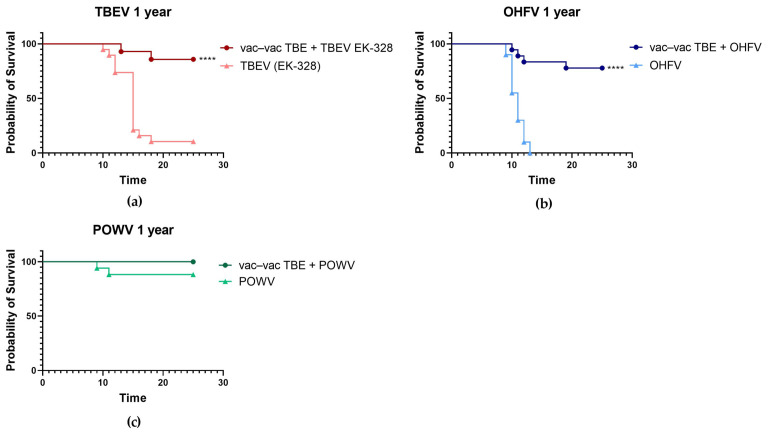
The survival curves of mice infected with TBEV(EK-328) (**a**), OHFV (**b**), and POWV (**c**) at doses of 100LD_50_ one year after double-dose immunization with the TBE vaccine, *n* = 16–20. The statistical significance of the mortality data was determined using the log-rank test (**** *p* < 0.0001).

One year after two of the TBE vaccine doses in the group of mice infected with TBEV (EK-328) showed that the vaccine was highly effective in preventing both mortality and morbidity, compared to the control group of unvaccinated mice. Concurrently, the virus was detected in the CNS at 24–28 dpi in 21% of the surviving vaccinated animals, while this figure was 100% in the control group (Figure 8a, Table 5).

One year after double-dose immunization with the TBE vaccine, the survival rate and the absence of signs of the disease were observed in 78% of animals in the group infected with OHFV, vs. 0% in the control group that did not receive the vaccine. Hence, even one year after the last immunization, the vaccine showed high efficacy against OHFV infection (Figure 8b, Table 5).

Mice vaccinated and infected with POWV one year after immunization with two TBE vaccine doses did not die; the percentage of healthy animals was 71% compared to the control group, where the survival rate was 88% and the percentage of healthy animals was 6% (Figure 8c, Table 5). The virus was detected in the CNS at 24–28 days post-infection in only 29% of vaccinated animals, compared to 82% of unvaccinated animals. As noted above, this suggests that adult mice are less susceptible to lethal infection with POWV, although they do succumb to infection, with a potential transition to chronic disease. However, the TBE vaccine provided partial protection against the disease and prevented the virus from entering the CNS.

#### 2.4.6. NAb Titers After Infection with TBEV, OHFV, and POWV One Year After Vaccination Against TBE

No booster antibody response was observed in the event of the infection occurring one year after double-dose vaccination, like it was in the case one month and three months after the last immunization with the TBE vaccine. In contrast, on day 2 post-infection, the titer of NAbs against the vaccine strain tended to decrease.

**Figure 9:**
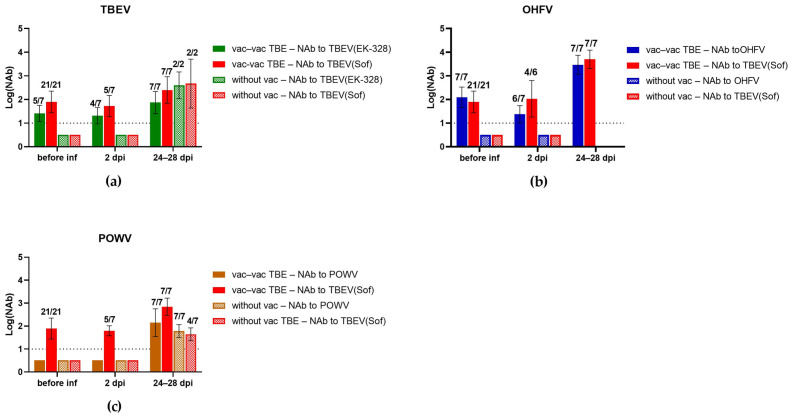
Mean geometric titers of NAbs in sera of seropositive mice against TBEV (EK-328) (**a**), OHFV (**b**), and POWV (**c**) one year after double-dose immunization with the TBE vaccine before and after infection with TBEV (EK-328), OHFV, and POWV. Vaccinated groups are shown as solid columns; unvaccinated control groups are shown as shaded columns. Unvaccinated mice infected with OHFV do not have a point at 24–28 days post-infection because of high mortality before the end of the experiment. The number of subjects in each group is as follows: N = 7 in the vaccinated group; N = 3 in the unvaccinated group before infection and at 2 days post-infection; N = 7 in the unvaccinated group infected with POWV; N = 2 in the infected with TBEV group at 24–28 days post-infection. The number of seropositive sera out of the total number of sera tested is indicated above each column. The dotted line indicates the presumed minimum protective titer of NAbs and the lower limit of NAb detection in PRNT_50_. In sera with NAb titers below 1 log, the conditional titer is taken as 0.5 log. The data are presented as mean ± SD.

One year post-vaccination, we analyzed the dynamics of the titers of NAbs against TBEV (EK-328) and the vaccine TBEV strain (Sofjin). In twice-vaccinated mice infected with TBEV (EK-328), the geometric mean titers of NAbs against TBEV strain Sofjin were higher than those against TBEV (EK-328) at all time points. Unvaccinated mice tended to have higher NAb titers for both the vaccine strain and TBEV (EK-328) at 24–28 days post-vaccination compared to vaccinated mice (Figure 9a).

In the group infected with OHFV, the mean geometric titers of NAbs against OHFV and NAbs against the vaccine TBE strain Sofjin still showed similar dynamics in vaccinated mice as was observed in mice infected three months after the last vaccination (Figure 9b).

NAbs against POWV in the sera of vaccinated mice were detected neither before nor two days post-infection; however, NAb titers to POWV had increased by 28 days post-infection (Figure 9c). In the group of mice twice vaccinated against TBE one year before and infected with POWV, the titers of NAbs against the vaccine strain were increased at all points. On the other hand, in groups of mice infected three months and one year after the last immunization with the TBE vaccine and infected with POWV, preliminary vaccination against TBE stimulated greater production of NAbs against POWV by days 24–28 compared to the unvaccinated control group. Unvaccinated mice infected with POWV had NAbs against TBEV (Sofjin), indicating high cross-reactivity among the antibodies produced.

#### 2.4.7. Prothrombin Time After Infection with TBEV, OHFV, and POWV

Since OHFV is characterized by hemorrhagic syndrome, this study aims to evaluate the blood coagulation system by measuring prothrombin time (PT), an indicator of the extrinsic blood coagulation pathway.

**Figure 10:**
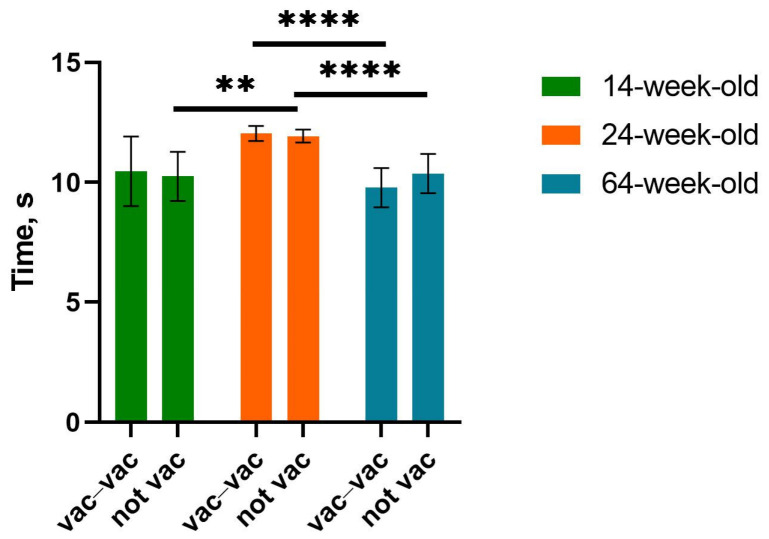
Prothrombin time at one month (green), three months (orange), and one year (blue) after immunization with TBE vaccine in vaccinated and unvaccinated mice. The spread is represented by the median value ± 95% CI, with statistical analysis using the Mann–Whitney U test (** *p* < 0.01, **** *p* < 0.0001).

First, in order to ascertain how age and vaccination affect the blood coagulation system in BALB/c mice, the PT was evaluated in mice of all ages, with and without prior immunization with the TBE vaccine (Figure 10). No significant differences were observed before and after immunization with the TBE vaccine, indicating that vaccination does not affect the PT. However, the PT was found to differ between age groups. The highest PT value was detected in the group of mature 24-week-old mice.

**Figure 11:**
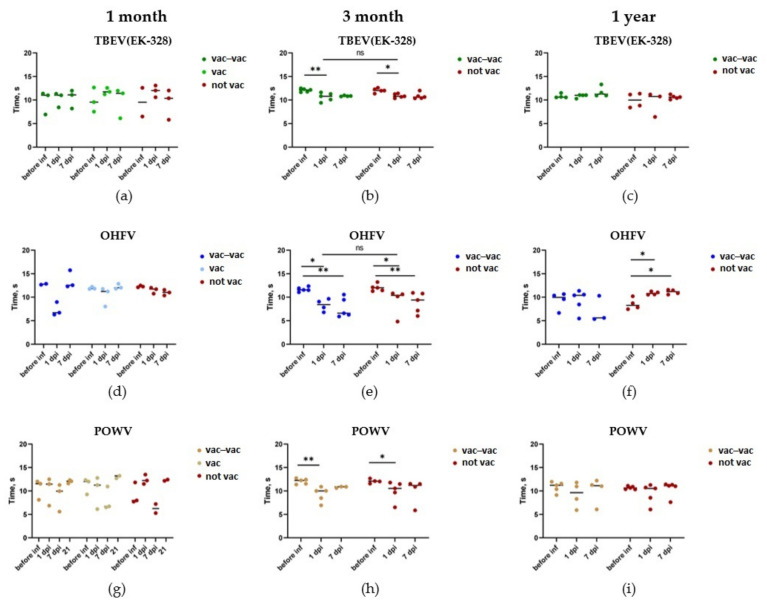
PT for mice infected with TBEV (EK-328) (**a**–**c**), OHFV (**d**–**f**), and POWV (**g**–**i**) one month (**a**,**d**,**g**)—young 14-week-old mice, three months (**b**,**e**,**h**)—mature 24-week-old mice, and one year (**c**,**f**,**i**)—old 64-week-old mice after vaccination against TBEV and without vaccination. Blood was taken from the same mice. “–” = median values, with statistical analysis using the Mann–Whitney U test (* *p* < 0.05, ** *p* < 0.01, ns—not significant).

In the absence of prior immunization, statistically significant changes in PT were observed only in the group of mature 24-week-old mice infected with each of the studied viruses (TBEV, OHFV, and POWV) three months after the start of the experiment and in the group of old mice infected with OHFV one year after the initiation of the experiment (Figure 11 b,c,e,h). A decline in PT one day post-infection with TBEV, OHFV, and POWV was observed. However, for OHFV-infected mice, the decline in PT persisted until seven days post-infection. In the group of older mice, PT increased on days one and seven post-infection. A downward trend in PT was also observed in the group of young POWV-infected mice seven days post-infection (Figure 11g). However, because of the limited sample size, the observed difference did not attain statistical significance.

For vaccinated animals, the decrease in PT was observed in groups of infected mature 24-week-old mice, similar to groups without prior immunization (Figure 11b,e,h). Differences in PT dynamics between vaccinated and unvaccinated mice were revealed in the group of older mice infected with OHFV (Figure 11f). In unvaccinated mice, PT increased, while in vaccinated mice, PT tended to decrease on day 7 post-infection, and these mice survived to the end of the experiment. Furthermore, in young mice infected with OHFV (Figure 11d), PT decreased after two vaccinations but remained unchanged in non-immunized and single-dose vaccinated mice.

These results show that pre-vaccination can influence the PT dynamics in the early stages of infection, but it depends on the virus and animal age.

## 3. Discussion

Vast regions within Asia and Europe are characterized by a high prevalence of TBEV, accompanied by associated foci of various orthoflaviviruses that are transmitted by ticks, such OHFV and POWV. A significant proportion of the population residing in these areas has developed immunity to TBEV, either as a result of vaccination programs or through prior natural exposure to the virus. In this study, we conducted in vivo experiments with durations of post-vaccination immunity to examine how existing immunity to the TBE vaccine can affect subsequent infection with other orthoflaviviruses. Furthermore, the progression of tick-borne orthoflavivirus infections in young, mature, and old mice without prior immunization was examined. This approach has made it possible to assess the effectiveness of the TBE vaccine against OHFV and POWV and confirm the absence of ADE.

It seems that sex plays a significant role in the development of the immune response to infection, but this is a separate issue that needs to be addressed in future studies. There are studies that suggest that sex hormones influence the development of the immune response [69]. Although some studies have indicated the existence of sex disparities in the susceptibility of mice to infections caused by orthoflaviviruses [70], data concerning the role of sex as a predisposing factor for tick-borne encephalitis in mice remains extremely limited. In the present study, in order to standardize the experiment, mice of a single sex (females) were used. Moreover, our earlier research demonstrated that both male and female BALB/c mice are equally susceptible to infection with the tick-borne encephalitis virus [58].

It is well-known that the elderly population is predisposed to orthoflavivirus infection and exhibits a protracted recovery period [66]. Furthermore, a lower level of B- and CD4+ T-cell activation was observed in older recipients compared to younger ones following vaccination [65]. In the older population of the C57BL/6 mouse model, infection with POWV caused increased mortality and rate alterations in the profile of induced cytokines. [71].

In the present study, we examined how infection of BALB/c mice caused by TBEV, OHFV, and POWV changes with age. The 24-week-old mice infected with TBEV exhibited higher survival rates and increased median survival time compared to the 14-week-old (young) or 64-week-old (old) ones. Furthermore, the PT in groups of uninfected 24-week-old mice was longer than that in younger and older mice. Additional studies are needed to confirm this correlation.

The present study revealed no correlation between the age of mice infected with OHFV and their sensitivity to the virus. This is likely to have resulted from the high virulence of the virus, which caused 100% mortality by day 14 across all the age groups.

The impact of age on the severity of POWV infection in mice was found to be most significant. As the mice aged, the mortality following infection with POWV decreased, reaching 0% in 64-week-old mice. Concurrently, disease incidence increased with age, and viral RNA was detected in the CNS in a larger number of animals infected with POWV. One of the causes of mortality in orthoflavivirus infection is T-cell response as a pathogenetic factor [72]. In this case, we suppose that as the mice aged, the activation of the CD8+ T-cell immune response was insufficient, which is why some of the mice survived and the virus was not eliminated from the CNS. This fact may attest to the age-related decrease in sensitivity of BALB/c mice to POWV, or a possible transition of the infection to a chronic form [73]. Meanwhile, the titers of NAbs against POWV in mice by 28 days after infection do not change significantly with age.

The present study examined the duration of protection provided by the TBE vaccine against OHFV and POWV. According to the results of our experiments, single-dose vaccination with the TBE vaccine ensured protection against infection with TBEV EK-328, partial protection against infection with OHFV, and neither seroconversion nor protection against infection with POWV. Two doses of the TBE vaccine were highly effective against TBEV strain EK-328 and OHFV infection, both three months and one year after the last immunization with the Sofjin vaccine. There was no booster response for groups of mice infected with OHFV, but the dynamics of NAbs against OHFV and the vaccine strain were comparable and had no significant differences.

In mice vaccinated twice, there was no seropositive serum to POWV in any age group. However, double vaccinination exhibited partial protection against the death of animals after infection with POWV one month and three months after the second vaccination. Because of the low sensitivity of old mice to the virus, it was impossible to assess the effectiveness of the vaccine against mortality one year after the last vaccination. However, the TBE vaccine protected all mice against the disease and virus entering the CNS. The partial protection provided by the TBE vaccine against POWV may be attributable to two factors. Firstly, the activation of the T-cell immune response induced by immunization. Secondly, the presence of non-neutralizing antibodies capable of inducing a cytopathic effect. Alternatively, the presence of neutralizing antibodies to POWV that were below the detection limit in PRNT_50_ may be responsible. The dynamics of NAbs among vaccinated and POWV-infected mice exhibited age-related variations. Three months and one year after the last vaccination, titers of NAbs against POWV in the sera of infected mice were detected 28 days post-infection, in contrast to the mice infected one month after vaccination, where NAbs against POWV were undetectable.

Therefore, we can speak of the phenomenon of original antigenic sin (OAS). This phenomenon means that in response to secondary infection, the proliferation of cross-reactive memory B-cells induced by primary infection having lower affinity for secondary antigens dominate [74,75]. OAS promotes the production of antibodies and protection against closely related viruses, as can be seen in our case. The infection was stopped in the early stages due to antibodies against the vaccine strain TBEV. Cross-reactive antibodies against OHFV and POWV are produced more slowly or are not synthesized at all, as in the case of POWV infection one month after vaccination. This effect can significantly complicate differential serodiagnostics, since ambiguous results can be obtained not only in ELISA, but even in neutralization reactions.

**Figure 12:**
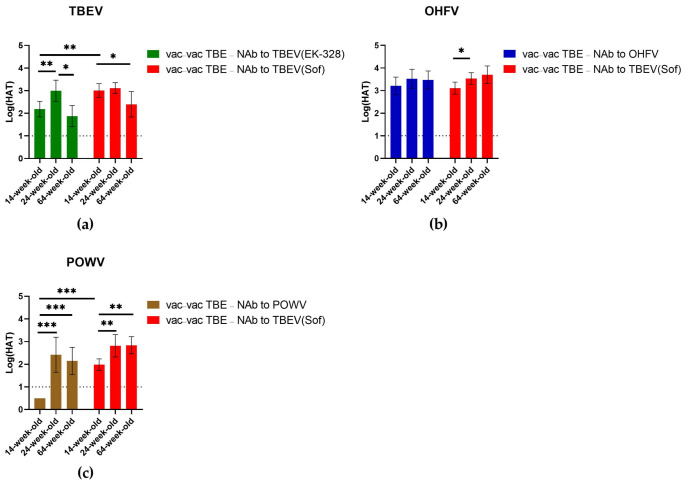
The mean geometric titers of NAbs on day 28 post-infection against TBEV strain EK-328 (green), TBEV vaccine strain Sofjin (red), OHFV (blue), and POWV (beige) for mice infected with TBEV (EK-328) (**a**), OHFV (**b**), and POWV (**c**) one month (14-week-old), three months (24-week-old), one year (64-week-old) after vaccination against TBEV. The dotted line indicates the estimated minimum protective titer of NAbs and the lower limit of NAb detection in PRNT_50_. The spread is represented by the mean value ± SD, with statistical analysis using the Mann–Whitney U test (* *p* < 0.05, ** *p* < 0.01, *** *p* < 0.001).

In a group of mice twice immunized with the TBE vaccine and infected with TBEV (EK-328), the titers of NAbs against TBEV (EK-328) at 28 days post-infection were lower in young and old mice compared to mature mice, while the titers against TBEV (Sofjin) were lower only in old mice. This indicates a difference in the spectrum of NAbs in mice of different ages (Figure 12a).

In the group of vaccinated animals infected with OHFV on day 28 post-infection, the titers of NAbs against the vaccine strain in young mice were lower than in the sera of mature and old mice (Figure 12b).

In the group of vaccinated mice infected with POWV, the titers of NAbs against POWV and the vaccine TBEV strain Sofjin on day 28 post-infection were statistically higher in mature mice infected three months after vaccination and older mice infected one year after vaccination than in young mice infected one month after vaccination (Figure 12c). It may indicate that with increasing time since vaccination, the spectrum of neutralizing antibodies induced by infection with a heterologous virus changes, as well as indicating age-related changes.

Laboratory mice are the most convenient and common model for studying orthoflaviviruses [76,77]. Nonetheless, there is a possibility that the spectrum of Abs in humans and mice differs, and experiments on monkeys are therefore needed to completely rule out ADE [78,79]. For instance, during orthoflavivirus infection in mice, antibodies directed against the dI+II domains of protein E, known to be cross-reactive and capable of being associated with ADE, are less abundant [80]. At the same time, during TBEV infection, antibodies to dIII and dI+II domains are detected in the serum of monkeys and humans [39,60].

Changes in the spectrum of antibodies depending on the time elapsed since vaccination or on age may be a cause of ADE. However, in none of our experiments did we observe signs of ADE, such as an increase in morbidity or mortality, or a reduction in life expectancy.

Since OHFV can cause a hemorrhagic effect even in a mouse model [76], we evaluated the changes in the coagulation system based on the PT. The PT test measures how long it takes for a fibrin clot to form. This test evaluates the extrinsic and common pathways of the blood coagulation system, focusing specifically on the activity of prothrombin complex factors (II, V, VII, and X). A prolonged PT may be associated with factor (II, V, VII, X) deficiency; the phase of hypocoagulation in disseminated intravascular coagulation (DIC) syndrome; hypofibrinogenemia; and liver damage. Shortening of PT may be associated with activation of the extrinsic and common coagulation pathways and the phase of hypercoagulation in DIC syndrome [81,82]. Tissue factor, which activates the blood coagulation pathway, can be induced in endothelial cells under the influence of proinflammatory cytokines [83], or by the stage of hypercoagulation in DIC syndrome, but this requires further investigation. Studies investigating the coagulation mechanisms in patients with dengue hemorrhagic fever and severe clinical manifestations during in-hospital treatment have shown that PT often increases [84]. It is associated with liver damage and/or variable reduction in activation of factors (II, V, VII, and X), which are involved in the extrinsic or common coagulation pathway [81,85].

The mechanisms underlying the haemorrhagic syndrome in OHF remain to be investigated. However, the findings of the present experiment demonstrate that the hypocoagulation characteristic of haemorrhagic fevers, associated with viral replication, is compensated for in young animals and poorly compensated for in older unvaccinated ones in the early stages of infection; nevertheless, both groups ultimately succumb to the infection. Vaccination, by virtue of its capacity to restrict viral replication, offers protection against mortality and hypocoagulation. The mechanisms of protection and the link to hypercoagulation remain to be investigated.

According to the results, PT in the early stages of orthoflavivirus infection depends on the age of the mice and may depend on vaccination; at the same time, more pronounced changes were observed in mice infected with OHFV.

In the present study, experiments on mice demonstrated that the inactivated TBE vaccine provides long-term protection against OHFV and partial protection against POWV without signs of ADE. Furthermore, our results show that the spectrum of antibodies induced by the TBE vaccine, as well as the antibodies produced following infection with other orthoflaviviruses, depends on the time elapsed since immunization and the age of the animals. These findings suggest that the risk of ADE cannot be ruled out; further research is required. The presence of the OAS effect was demonstrated to significantly impede the diagnosis of orthoflavivirus infections when an immune response to another orthoflavivirus is present. According to the findings reported in this study, as outlined in the experiments conducted [39], the emergence of antibodies to the TBE vaccine at the onset of POWV and OHFV infection shows that even a neutralization reaction can give a false positive result for diagnosis. The presented data indicate that innovative methodologies are needed for developing diagnostic test systems for differential serodiagnostics, especially in regions where multiple viruses may be circulating.

## 4. Materials and Methods

### 4.1. Cells and Viruses

Porcine embryo kidney (PEK) cell lines were maintained at 37 °C in medium 199 with Hanks’ balanced salt solution and Earle’s balanced salt solution (2:1, *v*:*v*, (Chumakov FSC R&D IBP RAS (Institute of Poliomyelitis), Moscow, Russia)) supplemented with 5% fetal bovine serum (FBS, Gibco, New York, NY, USA). The viruses are listed in Table 6.

### 4.2. Animals

Female susceptible inbred BALB/c mice (the Stolbovaya branch of the Scientific Center for Biomedicine Technologies of the Federal Medical and Biological Agency of the Russian Federation) were used in this study. At the time of vaccination, the mice were 8 weeks old. Each group consisted of 15 to 20 animals, which is the optimal number for statistical analysis. Mice that were deemed to be unhealthy or had a weight of less than 12 g were excluded from the study. The mice were grouped according to their weight, with randomization employed to ensure an equitable distribution. The animals were maintained in accordance with the international guidelines for the handling of laboratory animals (CIOMS Recommendations, 1985, Directive 2010/63/EC and Annex A of the European Convention ETS No. 123). The study protocol was approved by the Ethics Committee of the Chumakov FSC R&D IBP RAS (Institute of Poliomyelitis), protocol No. 210121—4 January 2021.

### 4.3. Vaccination and Infection

**Figure 13:**
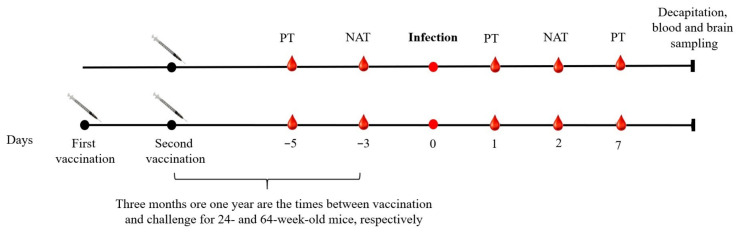
Experimental design. The syringe indicates vaccination; the red drop indicates blood sampling for PT (prothrombin time) and NAbs (neutralizing antibodies).

Immunization was performed using the whole-virion formaldehyde inactivated Tick-E-Vac vaccine (Chumakov FSC R&D IBP RAS (Institute of Poliomyelitis), Moscow, Russia). The vaccine was administered intramuscularly in the thigh, with 50 µL (vaccine antigen 0.1 μg per mouse [86]) either once or twice, with a two-week interval between each injection. One month, three months, and one year after vaccination, corresponding to 14 (15 mice to each group), 24 (20 mice to each group), and 64 weeks of age (20 mice to each group), mice were subcutaneously injected with 100 µL of virus prediluted in medium 199 in Earle’s salts (Chumakov FSC R&D IBP RAS (Institute of Poliomyelitis), Moscow, Russia) (Figure 13). The animals were infected with a dose of 100LD_50_ for each virus. Unvaccinated mice were also infected with the virus at the same time as the vaccinated mice. A total of 21 groups of mice were included in the study. Blood samples were taken from the submandibular vein of mice on days 5 and 3 before infection and on days 1, 2, and 7 post-infection to measure the prothrombin time (PT) and NAb titer (Figure 13). After infection, mice were weighed daily for 24–28 days and monitored for clinical manifestations of the disease. Disease was determined by weight loss, hair ruffling, hunched posture, as well as paresis and paralyzes of the fore and hind limbs. At the end of the experiment, surviving mice were decapitated; blood and brain were collected for examination.

### 4.4. Plaque Assay and 50% Plaque Reduction Neutralization Test (PRNT50)

Virus titration and neutralization assay were performed in 24-well plates using PEK cell culture according to the procedure described previously [58]. Briefly, serial tenfold dilutions of the virus were incubated with PEK cells for 1 h at 37 °C in a CO_2_ incubator, coated with 1.26% methylcellulose (Sigma, St. Louis, MO, USA) in medium 199 with Hanks’ and Earle’s salts (2:1, *v*:*v*) containing 2% FBS (Gibco, New York, NY, USA), and incubated in a CO_2_ incubator at 37 °C for 6 days. The cells were then fixed in 96% ethanol and stained with 0.4% solution of gentian violet dye (Alfa Aesar, Ward Hill, MA, USA). Virus titer was counted according to the Reed–Muench method and expressed as the logarithm of plaque-forming units per ml (logPFUs/mL) [87].

To determine the NAb titer in plaque reduction neutralization tests (PRNT_50_), dilutions of sera were made in medium 199 with Earle’s salt supplemented with 2% FBS (Gibco, New York, NY, USA) and incubated with TBEV, POWV, and OHFV in a 1:1 ratio for 1 h at 37 °C (the virus concentration being 30–40 PFUs per well). Next, 100 µL of the virus–serum mixture was added to the PEK cells in 24-well plates, following the same procedure as described for the plaque assay. The lower limit of NAT detection is 1 log in PRNT_50_. Each experiment included a negative and a positive serum with the known antibody titer as a control. NAb titers were calculated as log(NAb) according to the modified Reed–Muench method [87].

### 4.5. Titration on Mice

For determining the 50% lethal dose of infection, virus titration was performed in BALB/c mice prior to the first infection, when the mice weighed 14–16 g. Mice in each group (five mice per group) were subcutaneously injected with 100 µL of tenfold dilutions of the virus in medium 199 with Earle’s salt, and monitored for 21 days for clinical manifestations, symptoms, and mortality. The lethal dose of the virus resulting in 50% mortality was calculated using the Kärber method and expressed as logLD50/mL [88].

### 4.6. Virus RNA Quantification (qRT-PCR) in the Mouse Brain

Mouse brain samples were homogenized, and 10% brain suspensions in medium 199 with Earle’s salt were prepared. The samples were then stored at −70 °C. Viral RNA was isolated from 0.125 mL of 10% brain suspension using TRI Reagent LS (Sigma, USA) according to the instructions. Before RNA isolation, a fixed amount of attenuated vaccine strain of Sabin type 1 poliovirus was added to each sample as an internal control. Reverse transcription (RT) was performed using M-MLV revertase (Promega, Madison, WI, USA) according to the attached protocol. Primers for RT and quantitative RT-PCR (qRT-PCR) are listed in Table 7. Standards for qRT-PCR on TBEV and POWV were prepared according to the procedure described earlier [89]. Quantitative RT-PCR was performed on a C1000 thermocycler with a Chromo4 detector (BioRad, Hercules, CA, USA) using the RT-PCR reagent kit (Synthol, Moscow, Russia). The limit of detection for qRT-PCR was determined by serial dilutions of the standards, and was 100 RNA copies for TBEV and POWV per sample. The presence of viral RNA for OHFV was detected by cycle; a sample was considered positive if it became detectable earlier than cycle 32 of PCR.

### 4.7. Prothrombin Time

Blood was collected into tubes containing 3.8% trisodium citrate (Eco-Service, Saint Petersburg, Russia) at a ratio of 9:1 from the submandibular vein (3–5 mice in each group) before infection, on the day after infection, and on day 7 post-infection for determining PT. PT was counted by employing a non-automated method using the Diagem-P kit (Renam, Moscow, Russia), according to the instructions.

### 4.8. Statistical Analysis

Statistical analyses were performed using the GraphPad Prism 9 software (GraphPad Software Inc., San Diego, CA, USA). For survival analysis, Kaplan–Meier curves were constructed and analyzed using the log-rank test with Bonferroni correction for multiple comparisons. The neuroinvasiveness index was calculated using the formula (logPFU-logLD_50_). The titers of NAb, PT, and median life expectancy were analyzed using the Mann–Whitney U test.

## Figures and Tables

**Table 1:** The neuroinvasiveness index and virus doses used in infection.

Virus	Neuroinvasiveness Index (log(PFU/mL) − log(LD_50_/mL))	Doses for Infection	
		LD_50_	log(PFU)
TBEV	1.0 ± 0.6 *	100	3.4
OHFV	1.6 ± 0.6 *	100	3.8
POWV	2.8 ± 0.6 *	100	4.6

* 0.6—the method error determined in a separate experiment.

**Table 2:** Characteristics of orthoflavivirus infections caused by TBEV, OHFV, and POWV at doses of 100LD_50_ * in experiments on BALB/c mice of different age.

Challenge Virus	Age of Animals, Weeks	Number of Animals	Median Life Expectancy, Day [Min; Max]	Mortality, %	Morbidity, %	Number of Mice with Virus in the CNS, % **
TBEV (EK-328)	14	15	14 [8; 16]	87	100	87
	24	20	17 [11; 25] ***^a^	75	95	100
	64	19	14 [9; 17]	89.5	100	100
OHFV	14	15	10 [9; 13]	100	100	100
	24	20	9 [7; 11]	100	100	100
	64	20	10 [8; 12]	100	100	100
POWV	14	15	10 [7; 15]	60	60	67
	24	20	8 [7; 11]	60	90	70
	64	17	9 [8; 10]	12	94 ***^b^	82

* A 100LD_50_ was determined in young 14-week-old mice. ** The virus in the CNS of dead and surviving animals by 24–28 days post-infection by RT-PCR. *** Statistically significant difference from 14-week-old mice according to (^a^) the Mann–Whitney test (*p* < 0.05) and (^b^) Fisher’s exact test (*p* < 0.05).

**Table 3:** Characteristics of the infections caused by TBEV, OHFV, and POWV at doses of 100 LD_50_ * one month after single- and double-dose immunization with the TBE vaccine (Sofjin) in an experiment on BALB/c mice.

Challenge Virus	Number of Mice	First Vaccination	Second Vaccination	Median Life Expectancy, Days [Min; Max]	Survivors, %	Healthy, %	Number of Mice Without Virus in the CNS, % **
TBEV (EK-328)	15	−	−	14 [8; 16]	13	0	13
	15	−	+		93	93	93
	15	+	+		100	100	100
OHFV	15	−	−	10 [9; 13]	0	0	0
	15	−	+	10 [8; 13]	53	53	53
	15	+	+		100	100	100
POWV	15	−	−	10 [7; 15]	40	40	33
	15	−	+	14 [8; 15]	47	33	47
	15	+	+		100	100	100

* A 100LD_50_ was determined in young 14-week-old mice. ** It was assumed in this study that the virus had penetrated into the CNS of deceased mice. The virus identification was done in the CNS of surviving mice on 24–28 days post-infection. «+»—there is vaccination, «−»—no vaccination.

**Table 4:** Characteristics of infections caused by TBEV, OHFV, and POWV at doses of 100LD_50_ * three months after double-dose immunization with the TBE vaccine (Sofjin) in an experiment on BALB/c mice.

Challenge Virus	Number of Mice	Vaccination	Median Life Expectancy, Days [Min; Max]	Survivors, %	Healthy, %	Number of Mice Without Virus in the CNS, % **
TBEV (EK-328)	20	−	17 [11; 25]	25	5	0
	20	+	[17]	95	90	90
OHFV	20	−	9 [7; 11]	0	0	0
	20	+	[11; 13]	90	90	90
POWV	20	−	8 [7; 11]	40	10	30
	19	+	8 [7; 8]	79	79	79

* A 100LD_50_ was determined in young 14-week-old mice. ** It was assumed in this study that the virus had penetrated into the CNS of deceased mice. The virus identification was done in the CNS of surviving mice on 24–28 days post-infection. «+»—there is vaccination, «−»—no vaccination.

**Table 5:** The characteristics of the infections caused by a 100LD_50_ * of TBEV, OHFV, and POWV one year after the double-dose immunization with the TBE vaccine (strain Sofjin) in an experiment on BALB/c mice.

Challenge Virus	Number of Mice	Vaccination	Median Life Expectancy	Survivors, %	Healthy, %	Number of Mice Without Virus in the CNS, % **
TBEV (EK-328)	19	−	14 [9; 17]	10.5	0	0
	14	+	[17]	86	65	79
OHFV	20	−	10 [8; 12]	0	0	0
	18	+	10.5 [9; 18]	78	78	78
POWV	17	−	[12;14]	88	6	18
	18	+		100	71	71

* A 100LD_50_ was determined in young 14-week-old mice. ** It was assumed in this study that the virus had penetrated into the CNS of deceased mice. The virus identification was done in the CNS of surviving mice on 24–28 days post-infection. «+»—there is vaccination, «−»—no vaccination.

**Table 6:** Viruses used in the experiment.

Virus	Strain	Region and Year of Isolation	Isolate Source	Passage History *	GenBank Accession Number
TBEV, Siberian subtype	EK-328	Estonia, 1972	*I. persulcatus*	M6C1M6C1	DQ486861.1
TBEV, Far Eastern subtype	SofjinKGG	Primorsky Krai, 1937	Brain of deceased TBE patient	MxC1M3C1	GU121963
POWV	Pow-24	Primorsky Krai, Russia, 1975	*I. persulcatus*	M4V1C1	MG652438.1
OHFV, subtype 1	Nikitina	Omsk Region, Russia, 1948	Blood of a patient with OHF	MxM3V1C1	GU290187

* M—passage in the brain of a suckling mouse (Mx—unidentified number of early passages before the virus was obtained by the laboratory), V—passage in Vero cell culture, C—passage in PEK cell culture.

**Table 7:** Primers for RT and quantitative RT-PCR (qRT-PCR).

**For RT**	TBEV	TBE/Pow3′: 5′-AGCGGGTGTTTTTCCGAGTC-3′
	POWV	Pow_681r: 5′-AGACCTTTTCCCCCTAGAT-3′
	OHFV	OHFV_Real_R: 5′-TGT-CGA-ACT-CGC-AAG-CTG-AT-3′
	Polio	PVR1: 5′-CGAACGTGATCCTGAGTGTT-3′
**For qRT-PCR (forward, reverse, probe)**	TBEV	F-TBE1: 5′-GGGCGGTTCTTGTTCTCC-3′ R-TBE1: 5′-ACACATCACCTCCTTGTCAGACT -3′ TBE-Probe: 5′-(FAM)-TGAGCCACCATCACCCAGACACA-(BHQ1)-3′
	POWV	PowtestR2: 5′-CGTGACGCAAGAGTAGGTGA-3′ PowTestF: 5′-CCTTCACATGAGAGGGCGTC-3′ PowTestProbe: 5′-(R6G)GCGGGCCAGTGGAAGGGACGC(BHQ2)-3′
	OHFV	OHFV_Real_R: 5′-TGT-CGA-ACT-CGC-AAG-CTG-AT-3′ OHFV_Real_F: 5′-ATG-CTG-GTC-TTG-GGA-ACG-AG-3′ OHFV_probe: 5′-(FAM) GC-ACC-CAG-ACC-TGG-CCG-ATG (BHQ1)-3′
	Polio	PVR1: 5′-CGAACGTGATCCTGAGTGTT-3′ PVL1: 5′-GGCAGACGAGAAATACCCAT-3′ PVP1:5′-(R6G)-TTGATTCATGAATTTCCTTCATTGGCA-(BHQ1)-3′

## Data Availability

The original contributions presented in this study are included in the article. Further inquiries can be directed to the corresponding author.

## Abbreviations

Ab	antibody
ADE	antibody-dependent enhancement of infection
CNS	central nervous system
DENV	dengue virus
dpi	days post infection
ELISA	enzyme-linked immunosorbent assay
JEV	Japanese encephalitis virus
KFDV	Kyasanur Forest disease virus
LD	lethal dose
NAb	neutralizing antibody
OAS	original antigenic sin
OHF	Omsk hemorrhagic fever
OHFV	Omsk hemorrhagic fever virus
POWV	Powassan virus
PT	prothrombin time
PFU	plaque-forming unit
TBE	tick-borne encephalitis
TBEV	tick-borne encephalit virus
WNV	West Nile virus
YFV	yellow fever virus
ZIKV	Zika virus
